# Componential cognitive correlates of wayfinding in 5- to 6-year-old children: perspective taking, working memory, and nonverbal reasoning

**DOI:** 10.3389/fpsyg.2026.1893376

**Published:** 2026-09-22

**Authors:** Ming Li, Luyao Wang, Wei He

**Affiliations:** 1 Normal College, Jishou University, Jishou, China; 2 School of Sports and Health, Guangzhou Sport University, Guangzhou, China

**Keywords:** children, nonverbal reasoning, perspective taking, wayfinding, working memory

## Abstract

**Introduction:**

Wayfinding in early childhood is often treated as a general ability to learn or reproduce routes, yet different navigation situations may rely on partially distinct cognitive processes. This study examined whether wayfinding in 5- to 6-year-old children shows component-specific performance patterns and cognitive correlates.

**Methods:**

Children completed three wayfinding tasks adapted from the Navigation Test Suite: route repetition, directional approach, and route retracing. They also completed measures of agent-based and non-agent-based visuospatial perspective taking (VSPT), maze-like perspective taking, forward and backward digit span, nonverbal reasoning, and receptive vocabulary. Performance differences across wayfinding tasks and their associations with cognitive measures were examined using repeated-measures comparisons, correlation analyses, and hierarchical regression models.

**Results:**

Children performed best on route repetition, followed by directional approach, and performed worst on route retracing. Correlations among the three wayfinding tasks were modest and selective. Hierarchical regression analyses further revealed component-specific cognitive correlates: agent-based VSPT uniquely predicted directional approach, whereas non-agent-based VSPT uniquely predicted route repetition. Backward digit span was associated primarily with directional approach, while nonverbal reasoning was associated with directional approach and route retracing.

**Discussion:**

The findings support a componential account of early wayfinding. Rather than reflecting a single route-learning ability, different wayfinding situations appear to draw on partly differentiated cognitive processes. In particular, the observed patterns point to potentially distinct contributions of route reproduction, reference-frame transformation, executive updating, and route-level spatial reconstruction to wayfinding during the late preschool period.

## Introduction

Wayfinding refers to the process of determining and following a route through an environment in order to reach a destination (Golledge, 1999; Montello, 2005). For children, this process is central to everyday spatial functioning: they must learn routes, recognize landmarks, judge directions, and adjust their movements in both familiar and unfamiliar environments. Yet children’s ability to wayfind should not be treated as a single capacity. Classic accounts of spatial development distinguish among different forms of spatial knowledge, including landmark, route, and survey knowledge (Piaget and Inhelder, 1967; Siegel and White, 1975). Although later work has challenged a strict stage-like sequence and suggested that these forms of knowledge may emerge in parallel and interact during learning (Montello, 1998), the componential view remains useful for identifying the distinct processes involved in wayfinding (Chrastil, 2013). Thus, treating children’s wayfinding as a global ability may obscure theoretically meaningful differences among its component processes.

If wayfinding consists of separable component processes, tasks that vary in route direction, approach direction, and route reconstruction should impose different cognitive demands. In adult navigation research, Wiener and colleagues developed the Navigation Test Suite to distinguish several related aspects of wayfinding, including route repetition, route retracing, and directional approach (Wiener et al., 2020). Route repetition requires individuals to reproduce a learned route in the original direction and primarily draws on egocentric route learning and landmark–action associations. Route retracing requires returning from the endpoint to the start, which involves reversing the learned sequence and updating turning relations. Directional approach requires judging a target or route relation from a new approach direction and therefore places greater demands on directional transformation and the reconfiguration of landmark relations. Adult studies further suggest that route repetition and route retracing, although both based on a learned route, differ in cognitive demands and strategy use (Wiener et al., 2012). More recent work has further shown that route repetition and route retracing may rely on different combinations of wayfinding strategies, with turn-sequence knowledge contributing more clearly to route repetition than to retracing (Wiener et al., 2024). Given the methodological heterogeneity of children’s wayfinding research in age groups, environments, exposure procedures, task formats, and outcome measures (Yang and Merrill, 2022), applying a structured task set to the same child sample offers a way to test whether these tasks show distinguishable performance profiles and task-specific cognitive correlates in early childhood.

This question is particularly important for 5- to 6-year-old children, who are moving from preschool into formal schooling. At this age, children can often learn and reproduce simple routes, but their wayfinding remains strongly constrained by landmark salience, landmark stability, and the encoding of landmarks at decision points (Cornell et al., 1992; Lingwood et al., 2015a). Recent developmental work further suggests that children’s wayfinding is linked to specific spatial and cognitive abilities rather than to a single general spatial skill. For example, small-scale spatial abilities selectively predict children’s route and landmark knowledge, and landmark-based wayfinding varies with working memory and left–right language production (Lasc et al., 2025; van Hoogmoed et al., 2022). At the same time, abilities involved in more flexible navigation, including left–right understanding, perspective transformation, and executive control, are still developing. Taken together, these findings suggest that early wayfinding is shaped not only by route memory but also by children’s ability to encode, transform, and reorganize landmark–direction relations. However, it remains unclear which cognitive abilities support which components of wayfinding during this developmental period.

Visuospatial perspective taking (VSPT) is one plausible mechanism underlying such flexibility. VSPT refers to the ability to represent spatial relations from a viewpoint other than one’s current position or orientation. It requires children to inhibit their own egocentric perspective, imagine a layout from another location or direction, and judge directional relations accordingly (Flavell et al., 1981; Frick et al., 2014). This ability is relevant to wayfinding because successful navigation requires continuous updating of relations among body orientation, landmarks, and target direction, rather than simple memory for a sequence of movements (Hegarty and Waller, 2005). More broadly, recent work on map reading suggests that spatial perspective taking is also involved in coordinating egocentric and allocentric reference frames, particularly when individuals transform allocentric spatial information into an egocentric action-oriented perspective during wayfinding (Ding et al., 2025; Fehringer et al., 2025). However, VSPT should not be expected to support all wayfinding components in the same way. Directional approach provides the clearest case in which perspective transformation should matter, because children must evaluate spatial relations from a new direction. Route repetition may rely less directly on VSPT, although young children may still need to coordinate landmarks, bodily orientation, and action direction when reproducing a route. Route retracing may be different again: it requires children to reverse route structure across multiple decision points rather than simply adopt a local alternative viewpoint.

If VSPT contributes differently to wayfinding components, it is also necessary to consider which form of VSPT is being measured. VSPT tasks can elicit different strategies, including embodied self-rotation, scene rotation, rule-based left–right reversal, and perspective computation from another person’s position (Samuel et al., 2024). One important distinction concerns whether an external agent is present. A visible body may provide an anchor for judging spatial relations from another viewpoint, but it may also introduce self-other interference or additional social-spatial processing. Studies with adults show that another person’s position can influence spatial judgments even when that perspective is not explicitly required (Tversky and Hard, 2009; Samson et al., 2010; Surtees et al., 2013). More recent eye-tracking evidence further suggests that agent–object relations can shape attention allocation and response times in level-2 visual perspective taking (Ford et al., 2024). Developmental evidence further suggests that agent cues do not necessarily facilitate children’s spatial updating. Li et al. (2025) found that children younger than 7 years performed better in a self-based spatial updating condition than in an agent-based condition, particularly when viewpoint disparities exceeded 90°, suggesting that social or agent-related processing may sometimes impose additional cognitive demands during perspective transformation.

Beyond VSPT, children’s wayfinding may draw on broader cognitive abilities. Previous research has shown that children’s route-learning performance in virtual environments can be associated with both spatial and verbal abilities (Merrill et al., 2016). Working memory may be particularly relevant because navigation requires children to maintain route information while updating direction across successive decision points. Nonverbal reasoning may also contribute because children must identify spatial relations among landmarks and reorganize route information under changing directional demands. Previous work has also shown that children’s landmark-based navigation is associated with working memory and varies according to the type of landmark information available during navigation (van Hoogmoed et al., 2022). Other route-learning studies have shown that children’s navigation improves when they are encouraged to attend to, or verbally label, landmarks at decision points, suggesting that route learning depends not only on spatial memory but also on attentional selection and language-supported encoding (Lingwood et al., 2015b).

Beyond VSPT, children’s wayfinding may draw on broader cognitive abilities, although existing evidence has mainly concerned broad route-learning outcomes or landmark-based wayfinding rather than component-specific tasks. Children’s route learning in virtual environments has been associated with spatial and verbal abilities (Merrill et al., 2016), and landmark-based wayfinding has been linked to working memory and left–right language production, particularly in egocentric tasks (van Hoogmoed et al., 2022). Route-learning studies also show that encouraging children to attend to or verbally label landmarks at decision points can improve performance, suggesting that children’s wayfinding involves attentional selection and language-supported encoding in addition to spatial memory (Lingwood et al., 2015b). These findings indicate that broader cognitive abilities may be relevant to children’s wayfinding, but they do not clarify whether such abilities are differentially associated with route repetition, directional approach, and route retracing. For example, Working memory may help children maintain and update route information across successive decision points, whereas nonverbal reasoning may support the flexible organization of spatial relations. Forward digit span primarily reflects short-term verbal maintenance, whereas backward digit span additionally involves information manipulation and executive updating (Baddeley, 2003; Wells et al., 2018). Receptive vocabulary was included because children’s performance may also depend on their understanding of verbal instructions and spatial terms.

## The present study

The present study examined whether three components of wayfinding show distinct performance profiles and cognitive correlates in 5- to 6-year-old children. Children completed directional approach, route repetition, and route retracing tasks from the Navigation Test Suite, along with agent-based VSPT, non-agent-based VSPT, a paper-and-pencil maze-like perspective-taking task, forward and backward digit span, Raven’s Progressive Matrices, and the Peabody Picture Vocabulary Test. The maze-like perspective-taking task was included as an additional small-scale measure of symbolic spatial transformation, and receptive vocabulary was included as a control for language comprehension. The study addressed three research questions:

RQ1. Do route repetition, directional approach, and route retracing show distinguishable performance profiles in 5- to 6-year-old children?

RQ2. Are agent-based and non-agent-based VSPT differentially associated with these wayfinding tasks?

RQ3. Do working memory and nonverbal reasoning show task-specific associations with wayfinding performance?

To answer the questions, we proposed the following hypotheses:

*H1*. The three wayfinding tasks would show distinguishable performance profiles, reflected in differences in performance levels and limited intertask overlap.

*H2*. VSPT would show task-specific associations, with agent-based VSPT expected to be most closely related to directional approach.

*H3*. Agent-based and non-agent-based VSPT would show partially distinct associations with wayfinding tasks.

*H4*. Working memory and nonverbal reasoning would be more strongly associated with tasks requiring directional updating or route reconstruction than with simple route reproduction.

## Method

### Participants

The present study recruited 5- to 6-year-old children (*n* = 84, girls = 40, *M_age_* = 5.31, SD = 0.47). Children were recruited from kindergartens in Guangzhou City, China. All participants were typically developing children. According to parent reports, none had known neurological disorders, severe visual or hearing impairments, or developmental disorders. All children were native speakers of Mandarin Chinese and were able to understand and complete the experimental instructions.

Before testing, written informed consent forms were distributed to children’s parents or legal guardians. The forms described the purpose of the study, testing procedures, confidentiality principles, and the child’s right to withdraw from the study at any time. Children also provided verbal assent before participating. The study was approved by the Ethics Committee of the authors university.

Because some children had missing or invalid data on individual tasks, the final sample size varied slightly across measures. All analyses were conducted using available data, and the specific sample size for each analysis is reported in the Results section.

## Measures and tasks

### Wayfinding tasks

The wayfinding tasks were adapted from the Navigation Test Suite developed by Wiener et al. (2020). The core structure of each task was retained, but the procedures were modified for 5- to 6-year-old children: instructions were simplified and delivered orally, four practice trials were administered before each task, and each task was shortened to 14 test trials to reduce fatigue and maintain engagement. The retained trials were selected to preserve variation in approach direction and to keep correct response locations approximately balanced. Task-specific training was completed immediately before the corresponding test phase, and the order of the three wayfinding tasks was randomized across children to reduce potential order effects. The three wayfinding tasks were administered in separate virtual environments with different routes and different landmark configurations.

### Directional-approach task

This task was designed to assess children’s ability to use landmark configurations to judge an originally encoded direction from a changed viewpoint. In each trial, children first watched a small car move toward and stop at a four-way intersection. They were asked to remember the direction from which the car had arrived and where the car was located. The scene then changed so that the child appeared to return to the same intersection from a different direction. From this new approach direction, children were asked to indicate which way they should go to find the car again (see Figure 1). The task included 14 trials. Each correct response was awarded 1 point, yielding a maximum score of 14.

**Figure 1:**
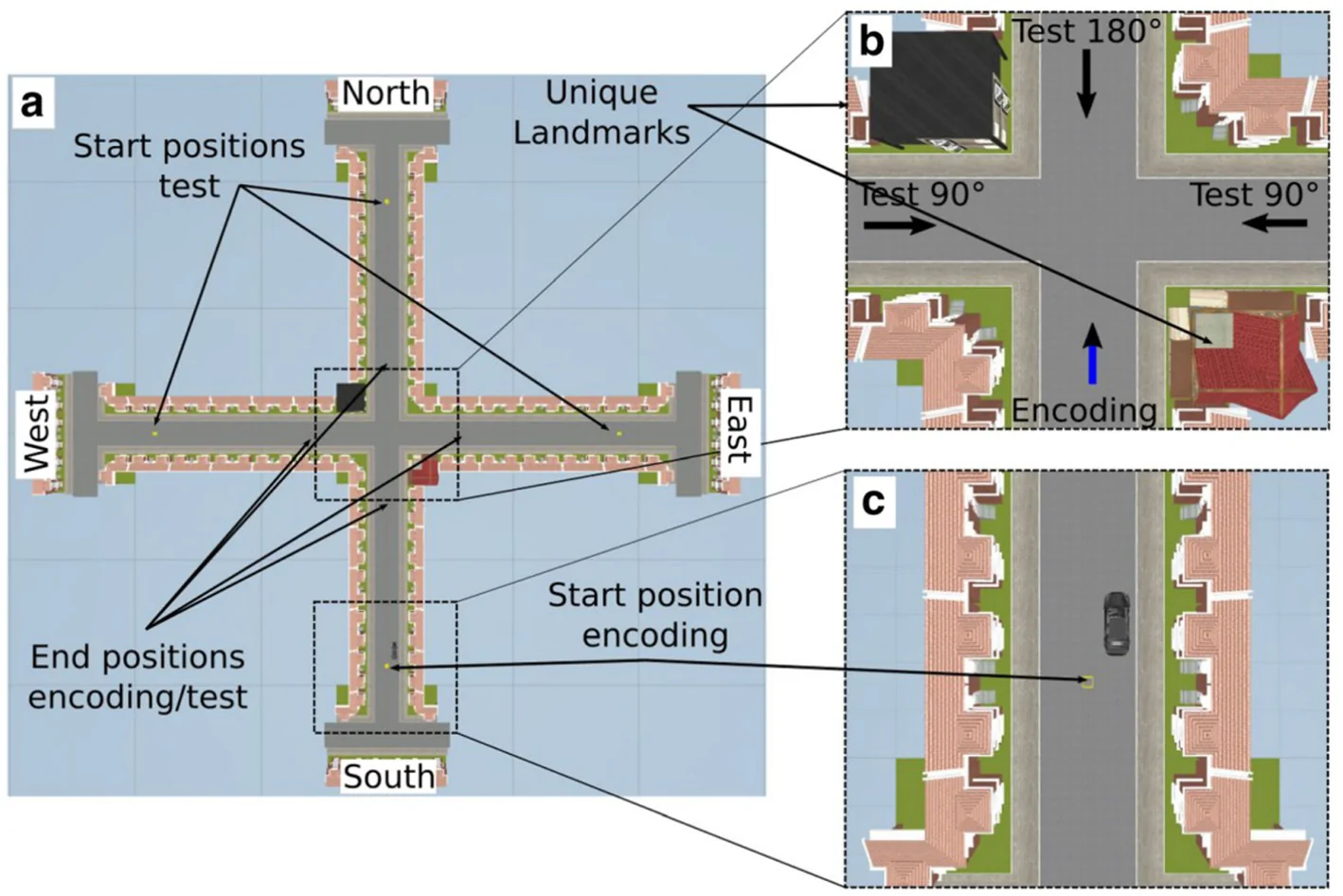
Schematic illustration of the directional-approach task. **(a)** Overview of the virtual environment showing the encoding and test start positions, encoding/test endpoints, and unique landmarks. **(b)** Top-down view of the intersection showing the encoding direction and alternative test approach directions (90° and 180°). **(c)** Example view during the encoding phase as the car approached the intersection. During encoding, children remembered the car’s location and its approach direction. During the test phase, they approached the same intersection from a different direction and indicated the direction in which the car had been located. The test approach and correct response directions were balanced across trials. Adapted from Wiener et al. (2020), distributed under the Creative Commons Attribution 4.0 International License (CC BY 4.0).

### Route-repetition task

The route-repetition task was used to assess children’s ability to reproduce a route in the original learning direction. In the learning phase, children watched a small car travel from a starting location to an endpoint through a sequence of intersections. In the test phase, children were placed back at the starting location and asked to repeat the same route in the original learning direction. At each intersection, the car stopped and children indicated which way the car should go next. After each response, the task continued to the next intersection along the route, regardless of whether the child’s response was correct or incorrect; thus, children did not receive corrective feedback during the test phase (see Figure 2). The task included 14 decision points. Each correct directional choice was awarded 1 point, yielding a maximum score of 14.

**Figure 2:**
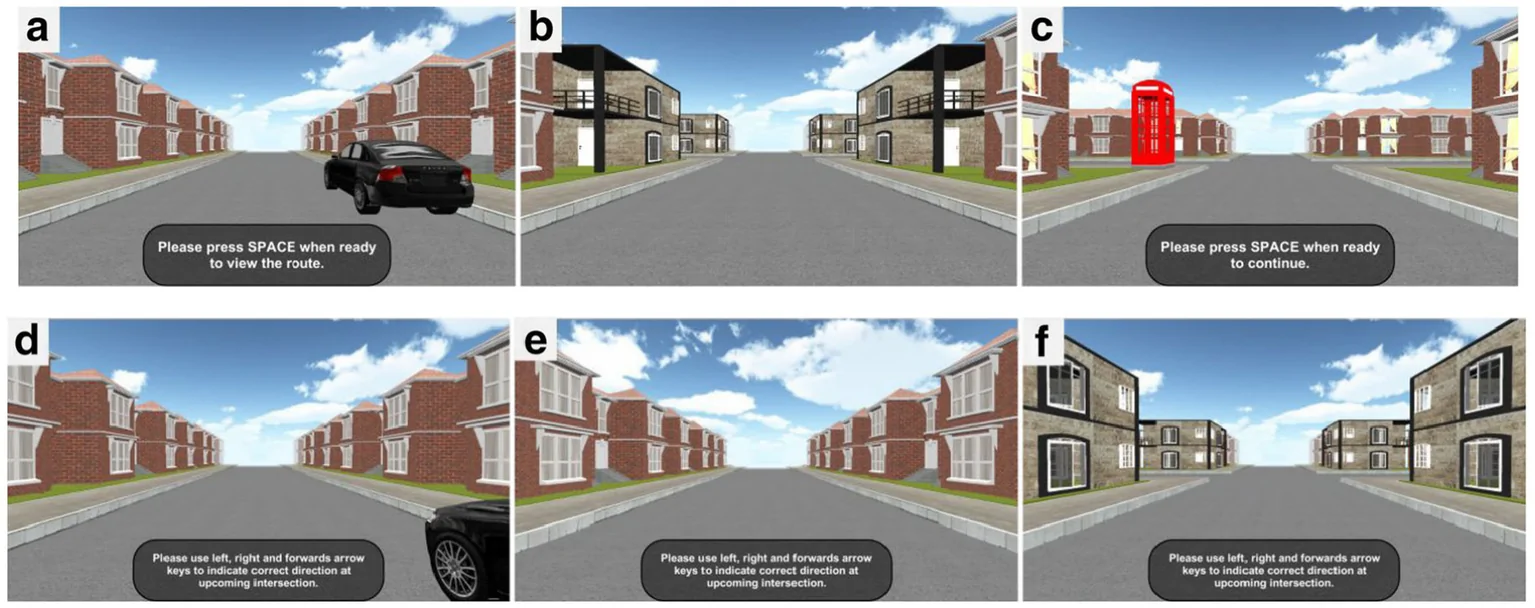
Example screenshots from the route-repetition task and route-retracing task. Learning phase. Children watched a small car travel passively from the starting point **(a)** to the endpoint **(c)** along a route with multiple intersections **(b)**. The car moved through each intersection in the correct direction, and children were asked to remember the route. Test phase (Route-Repetition Task). Children started again from the original starting point **(d)** and guided the car along the learned route in the same direction by choosing the correct turn at each intersection **(e, f)**. Test phase (Route-Retracing Task). The procedure was identical, except that children started from the endpoint and guided the car back to the original starting point in the reverse direction.

### Route-retracing task

The route-retracing task was used to assess children’s ability to return from the endpoint to the starting point along the learned route. The learning phase was identical to that of the route-repetition task: children watched a small car travel from a starting location to an endpoint through a sequence of intersections. In the test phase, children started from the endpoint and were asked to guide the car back to the original starting location along the learned route. At each intersection, the car stopped and children indicated the direction in which the car should continue. The task proceeded to the next intersection after each response, regardless of whether the response was correct or incorrect, and no corrective feedback was provided (see Figure 2). The task contained 14 decision points. Each correct directional choice was awarded 1 point, yielding a maximum score of 14.

### Visuospatial perspective-taking tasks

The present study included three visuospatial perspective-taking tasks to examine children’s ability to understand visuospatial relations from another person’s or another spatial position’s perspective.

### Agent-based VSPT task

The agent-based visuospatial perspective-taking task was adapted from the Photographic Perspective Taking Task (PPTT; Plummer et al., 2016). On each trial, children viewed a spatial display in which a small figure was seated on a chair and faced a set of objects. Below the display, several response options showed the same object arrangement from different viewpoints (see Figure 3). Children were asked to select the option that best represented what the figure would see from its seated position. Thus, the task required children to mentally transform the spatial layout from their own viewpoint to the external bodily viewpoint indicated by the agent.

**Figure 3:**
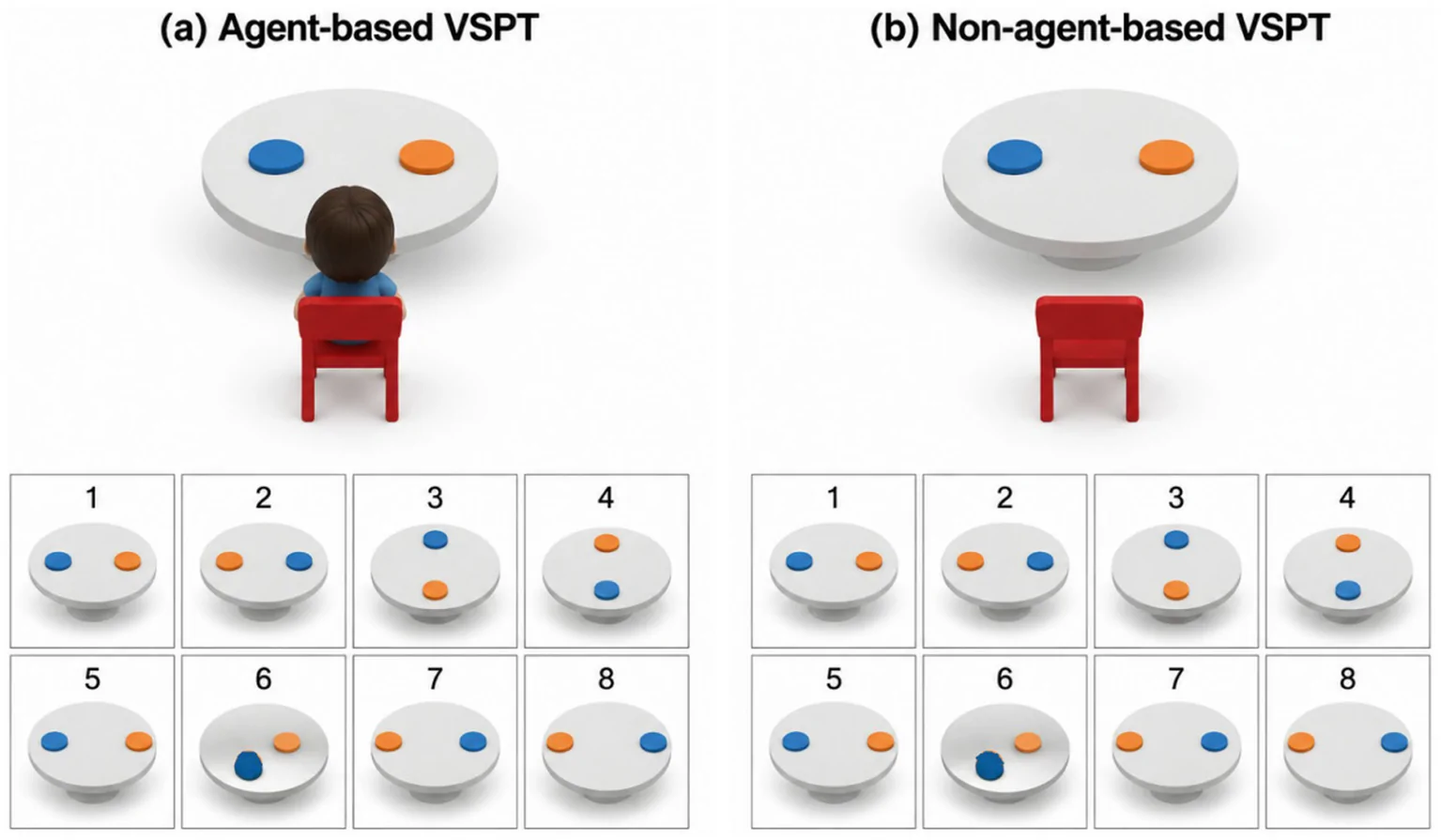
Schematic illustration of the agent-based and non-agent-based VSPT tasks. The task paradigm was adapted from Plummer et al. (2016). The illustration was created by the authors for explanatory purposes and does not reproduce the original test stimuli.

The task included eight possible agent orientations around the display, separated by 45° increments. Each orientation was presented twice, resulting in 16 test trials. Each correct response was awarded 1 point, yielding a maximum score of 16. Higher scores indicated better agent-based visuospatial perspective-taking ability.

### Non-agent-based VSPT task

The non-agent-based visuospatial perspective-taking task was structurally identical to the agent-based VSPT task. The spatial layout, object arrangement, chair position, viewpoint orientations, and response format were the same across the two tasks. The only difference was that, in the non-agent condition, no small figure was placed on the chair (see Figure 3). Children were still asked to select the response option that represented the spatial layout from the viewpoint indicated by the chair position and orientation.

Thus, this task required children to perform the same visuospatial transformation as in the agent-based task, but without an explicit external bodily agent. It was therefore used to assess children’s ability to transform spatial perspectives based on an abstract viewpoint cue rather than an embodied agent cue. The task included eight possible viewpoint orientations around the display, separated by 45° increments. Each orientation was presented twice, resulting in 16 test trials. Each correct response was awarded 1 point, yielding a maximum score of 16.

### Paper-and-pencil maze-like VSPT task

The paper-and-pencil maze-like VSPT task was adapted from Broadbent et al. (2014). Children were presented with a two-dimensional winding path on a sheet of paper and were asked to imagine themselves walking along the path from the start point to the finish point. At each turn, they indicated whether they would turn left or right from the imagined walking direction. Thus, the task required children to update their imagined heading within the path and make left–right directional judgments from the current perspective. Children were not allowed to rotate the paper during the task. The task included 20 turns, and each correct response was awarded 1 point, yielding a maximum score of 20 (see Figure 4).

**Figure 4:**
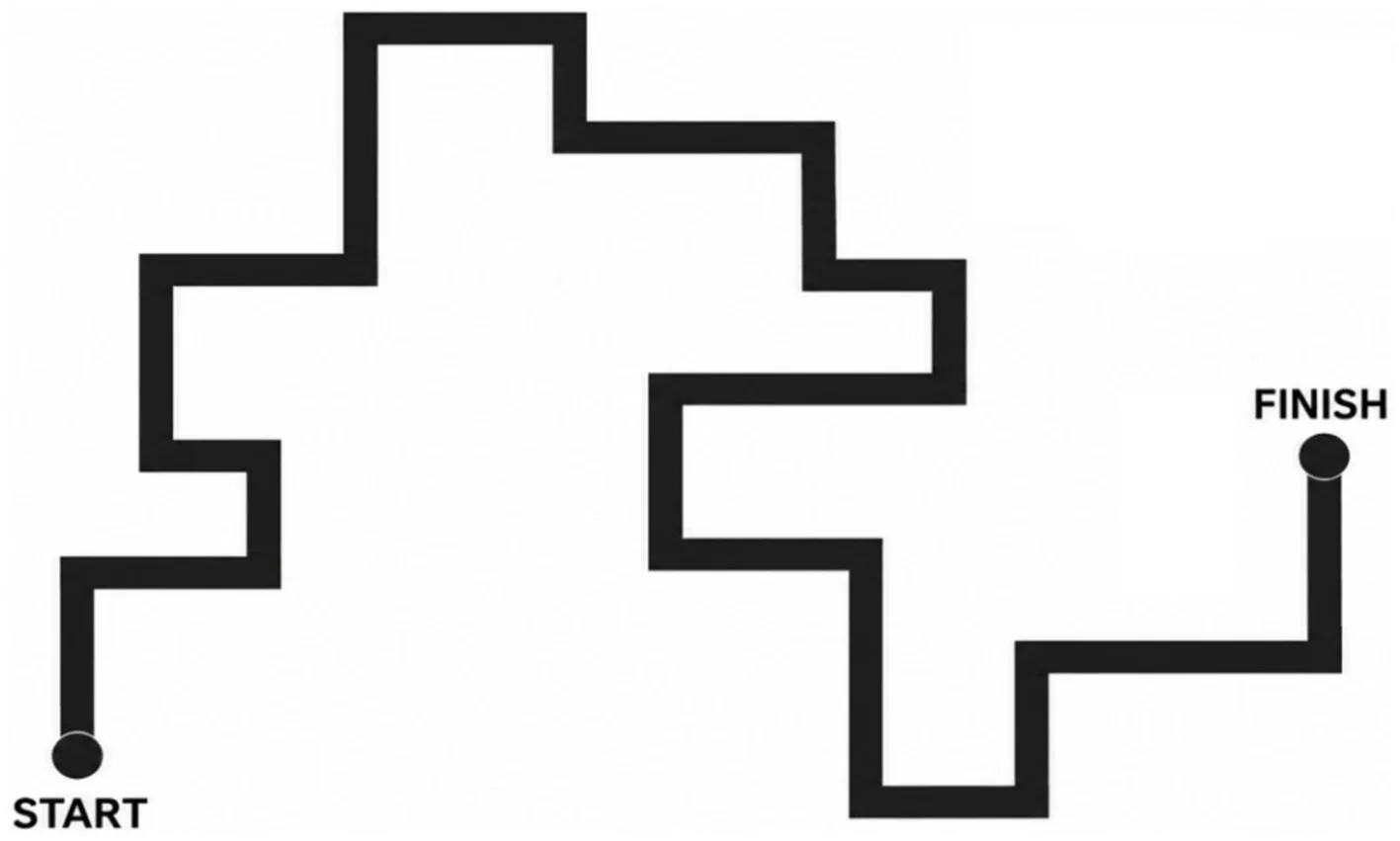
Paper-and-pencil maze-like VSPT task.

### Language ability

Children’s receptive language ability was assessed using the Chinese version of the Peabody Picture Vocabulary Test–Revised (PPVT-R; Sang and Miao, 1990; hereafter, PPVT). In each trial, the experimenter read a word aloud, and children were asked to select the picture that best matched the spoken word from a set of alternatives. The raw score was used in the analyses, with higher scores indicating better receptive vocabulary.

### Nonverbal reasoning

Children’s nonverbal reasoning ability was assessed using the Combined Raven’s Test, 2nd edition (CRT; Gao et al., 1998; hereafter, Raven). In this task, children were presented with visual matrices or figure patterns and were asked to select the missing figure that completed the pattern. The task primarily measures nonverbal reasoning, pattern detection, and relational reasoning. The raw score was used in the analyses, with higher scores indicating better nonverbal reasoning ability.

### Working memory tasks

Children’s forward and backward working memory were assessed using digit span tasks. In the forward digit span task, children were asked to repeat a sequence of digits in the same order as presented by the experimenter. The task included 24 items, with digit span lengths ranging from 2 to 9 digits and three items at each span length. In the backward digit span task, children were asked to repeat the digits in the reverse order. The task included 18 items, with digit span lengths ranging from 2 to 7 digits and three items at each span length.

### Testing procedure

All children were tested individually in a familiar room at their kindergarten. Testing was conducted by trained researchers who had received standardized instruction in the administration of all tasks. Before formal testing began, the testers visited the kindergarten 2 weeks in advance to establish rapport with the children.

Each child took approximately 60–70 min to complete all tasks. To reduce fatigue, testing was divided into two or three sessions when necessary, depending on the child’s state and level of engagement. Short breaks were provided between tasks.

Before the testing session, the experimenter first engaged in brief interaction with the child to ensure that the child understood the task rules and was willing to participate. Children then completed the tasks in a predetermined order. To minimize potential order effects, the presentation order of the wayfinding tasks and cognitive tasks was counterbalanced. Practice trials were provided for all tasks, and children proceeded to the formal test trials only after demonstrating that they understood the practice items.

For the wayfinding tasks, the experimenter introduced the route-learning and test rules using standardized instructions. The tasks were administered on a computer. At each decision point, children selected their response on the computer, and the program automatically recorded their choices and response accuracy. The visuospatial perspective-taking tasks, Raven’s Progressive Matrices, PPVT, and working memory tasks were scored according to their respective scoring rules.

### Data processing and analysis plan

Data analyses were conducted using JASP. First, descriptive statistics were computed for all main variables, including the mean, standard deviation, skewness, kurtosis, and valid sample size. The distributions of all variables were then examined, along with patterns of missing data and potential outliers. Because some children were absent during one or more testing sessions, they did not complete all tasks. As a result, valid sample sizes varied across analyses. Descriptive statistics and zero-order correlations were computed using available data, whereas hierarchical regression analyses used complete cases for the variables included in each model. To examine the missing-data mechanism, Little’s MCAR test was conducted across the main study variables. The test was not significant, *χ*^2^(45) = 54.888, *p* = 0.148, indicating no evidence that the missingness pattern systematically deviated from missing completely at random. The exact sample size for each analysis is reported in the Results section and relevant tables.

## Results

### Descriptive statistics and zero-order correlations

Before testing the research questions, descriptive statistics were first computed for the main variables, and Pearson zero-order correlations were calculated without controlling for covariates. The main variables included age, gender, PPVT, Raven’s Progressive Matrices, forward digit span, backward digit span, agent-based VSPT, non-agent-based VSPT, paper-and-pencil maze-like VSPT, and the three wayfinding tasks (Pairwise available *Ns* = 58–84, see Table 1). Means, standard deviations, and zero-order correlations for all variables are presented in Table 2. Zero-order correlations were conducted to provide a preliminary overview of the associations among age, gender, language ability, nonverbal reasoning, working memory, VSPT, and wayfinding performance. As shown in Table 1, the three wayfinding components showed distinguishable correlation patterns with the cognitive predictors.

**Table 1:** Sample sizes and missing-data handling for main analyses.

Analysis	Valid *N*	Missing-data handling
Descriptive statistics	75–84	Available data
Zero-order correlations	Pairwise available Ns 58–84	Pairwise available data
Repeated-measures ANOVA	75	Complete data on three wayfinding tasks
Directional approach regression	59	Complete cases
Route repetition regression	59	Complete cases
Route retracing regression	58	Complete cases

Valid Ns vary across analyses because some children did not complete all tasks. Correlations were computed using available cases; regression models used complete cases for variables entered in each model.

**Table 2:** Zero-order correlations among main study variables.

Variables	Mean (SD)	Age	Gender	PPVT	Raven	FDS	BDS	Agent-VSPT	Nonagent-VSPT	Maze-VSPT	DirApp	RouteRep	RouteRet
1. Age	5.31(0.47)	–											
2. Gender		0.135	–										
3. PPVT	87.52(24.95)	−0.150	−0.155	–									
4. Raven	28.70(7.13)	0.170	0.057	−0.103	–								
5. FDS	0.54(0.22)	−0.029	−0.022	0.602^***^	−0.264^*^	–							
6. BDS	0.20(0.11)	0.016	0.005	0.424^***^	0.186	0.282^*^	–						
7. Agent-VSPT	4.87(3.69)	0.199	−0.180	−0.156	0.276^*^	−0.208	0.111	–					
8. Nonagent-VSPT	5.26(4.53)	0.173	−0.182	0.027	0.124	−0.004	0.307^**^	0.775^***^	–				
9. Maze-VSPT	11.07(2.92)	−0.008	0.091	0.074	−0.078	−0.008	0.073	0.279^*^	0.361^***^	–			
10. DirApp	7.31(3.19)	0.103	−0.159	−0.301^*^	0.530^***^	−0.468^***^	0.244^*^	0.520^***^	0.316^**^	−0.010	–		
11. RouteRep	8.42(2.58)	0.085	−0.031	0.032	0.308^**^	−0.071	0.137	0.136	0.274^*^	0.141	0.115	–	
12. RouteRet	5.67(2.40)	−0.079	−0.298^**^	−0.173	0.263^*^	−0.040	−0.111	0.126	0.077	−0.073	0.243^*^	0.098	–

PPVT, Peabody Picture Vocabulary Test; FDS, forward digit span; BDS, backward digit span; VSPT, visuospatial perspective taking; DirApp, directional approach; RouteRep, route repetition; RouteRet, route retracing. Gender was coded as [1 = boys, 2 = girls]. ^*^p < 0.05, ^**^p < 0.01, ^***^p < 0.001.

For the VSPT measures, agent-based VSPT was significantly associated with directional approach, *r* = 0.520, *p* < 0.001, but was not significantly associated with route repetition, *r* = 0.136, *p* = 0.234, or route retracing, *r* = 0.126, *p* = 0.282. Non-agent-based VSPT was also associated with directional approach, *r* = 0.316, *p* = 0.005, and route repetition, *r* = 0.274, *p* = 0.015, but not with route retracing, *r* = 0.077, *p* = 0.510. These findings provide preliminary evidence that VSPT–wayfinding associations varied across task components, with directional approach showing the strongest association with agent-based VSPT and route repetition showing a more modest association with non-agent-based VSPT. The maze-like VSPT task was not significantly associated with any of the three wayfinding outcomes, *rs* = −0.073 to 0.141, *ps* ≥ 0.220.

Regarding broader cognitive abilities, Raven scores were significantly correlated with all three wayfinding outcomes: directional approach, *r* = 0.530, *p* < 0.001; route repetition, *r* = 0.308, *p* = 0.006; and route retracing, *r* = 0.263, *p* = 0.022. Backward digit span was significantly correlated with directional approach, *r* = 0.244, *p* = 0.036, but not with route repetition, *r* = 0.137, *p* = 0.244, or route retracing, *r* = −0.111, *p* = 0.358. Forward digit span showed a significant negative correlation with directional approach, *r* = −0.468, *p* < 0.001, but was not significantly related to route repetition, *r* = −0.071, *p* = 0.540, or route retracing, *r* = −0.040, *p* = 0.731. Forward digit span was strongly correlated with PPVT, *r* = 0.602, *p* < 0.001, suggesting that it may primarily reflect verbal sequential maintenance in this sample.

In addition, PPVT was negatively associated with directional approach, *r* = −0.301, *p* = 0.016, but was not significantly associated with route repetition or route retracing. Gender was significantly associated with route retracing, *r* = −0.298, *p* = 0.009, whereas age was not significantly associated with any wayfinding outcome, *rs* = −0.079 to 0.103, *ps* ≥ 0.370.

### Differences in children’s performance across wayfinding tasks

To examine whether 5- to 6-year-old children differed in performance across the three wayfinding components, a 3 × 2 mixed-design repeated-measures ANOVA was conducted, with task type as the within-subjects factor and gender as the between-subjects factor (*N* = 75, complete data on three wayfinding tasks). The three task types were directional approach, route repetition, and route retracing.

Mauchly’s test of sphericity was not significant, *W* = 0.956, *p* = 0.197, indicating that the assumption of sphericity was met. The analysis revealed a significant main effect of wayfinding task type, *F*(2, 146) = 25.41, *p* < 0.001, *η*^2^ = 0.165. The main effect of gender was also significant, *F*(1, 73) = 4.19, *p* = 0.044, *η*^2^ = 0.019. However, the task type × gender interaction was not significant, *F*(2, 146) = 1.22, *p* = 0.297, *η*^2^ = 0.008. Therefore, gender was treated as a control variable in subsequent analyses.

Bonferroni-corrected *post hoc* comparisons showed significant differences among all three tasks. Children performed best on route repetition (*M* = 8.42, SD = 2.58), followed by directional approach (*M* = 7.31, SD = 3.19), and showed the lowest performance on route retracing (*M* = 5.67, SD = 2.40). Specifically, performance on route repetition was significantly higher than performance on directional approach, MD = 1.18, *p* = 0.032, Cohen’s *d* = 0.44. Directional approach performance was significantly higher than route retracing performance, *MD* = 1.72, *p* < 0.001, Cohen’s *d* = 0.65. Route repetition performance was also significantly higher than route retracing performance, *MD* = 2.90, *p* < 0.001, Cohen’s *d* = 1.09.

RQ1 was supported. Children showed a clear performance gradient across the three tasks, with route repetition being easiest, directional approach intermediate, and route retracing most difficult. Intertask correlations were limited: only route repetition and route retracing were significantly correlated, and this association was modest, *r* = 0.243, *p* = 0.036. Thus, the results indicate that the three tasks differed in behavioral difficulty and showed limited overlap in performance. This pattern is consistent with the possibility that the tasks place partly different demands on children’s wayfinding, although it should not be taken as evidence for fully separable underlying components.

### Differential associations between VSPT and wayfinding components

Because the agent-based and non-agent-based VSPT tasks each included 16 four-choice trials, the expected chance score was 4. To examine whether children’s performance exceeded chance, one-sample t tests were conducted against this value. Performance was significantly above chance for both the agent-based VSPT task, *t*(83) = 2.16, *p* = 0.034, mean difference = 0.86, 95% CI [0.07, 1.67], and the non-agent-based VSPT task, *t*(83) = 2.55, *p* = 0.013, mean difference = 1.26, 95% CI [0.28, 2.25]. However, the mean scores were only modestly above chance, indicating that both tasks were difficult for this age group. A paired-samples t-test was conducted to compare children’s performance on the agent and non-agent VSPT tasks. To examine whether different forms of VSPT were associated with different wayfinding components, partial correlation analyses were first conducted. Hierarchical regression analyses were then performed to test the incremental predictive effects of agent-based and non-agent-based VSPT after controlling for age, gender, PPVT, Raven’s Progressive Matrices, forward digit span, and backward digit span.

### Comparison of performance on agent and nonagent VSPT tasks

Because the difference scores between agent-VSPT and nonagent-VSPT significantly deviated from normality, *W* = 0.697, *p* < 0.001, a Wilcoxon signed-rank test was conducted (*N* = 84). Children’s scores did not differ significantly between the agent-VSPT task (*M* = 4.87, SD = 3.69) and the nonagent-VSPT task (*M* = 5.26, SD = 4.53), *W* = 527.00, *z* = −0.15, *p* = 0.885, matched rank-biserial *r* = −0.03. The paired-samples *t*-test yielded the same conclusion, *t*(83) = −1.25, *p* = 0.213, Cohen’s *d* = 0.14.

### Partial correlations

After controlling for age, gender, PPVT, Raven’s Progressive Matrices, forward digit span, and backward digit span, agent-based VSPT was significantly and positively correlated with directional approach, *r* = 0.312, *p* = 0.023, whereas the partial correlation between non-agent-based VSPT and directional approach was not significant, *r* = 0.088, *p* = 0.530.

In contrast, non-agent-based VSPT was significantly and positively correlated with route repetition, *r* = 0.277, *p* = 0.045, whereas the partial correlation between agent-based VSPT and route repetition was not significant, *r* = 0.019, *p* = 0.895. Neither form of VSPT was significantly correlated with route retracing, all *ps* > 0.05.

### Hierarchical regression analyses

To examine whether the two forms of VSPT explained unique variance in each wayfinding component, three hierarchical regression models were conducted, with directional approach, route repetition, and route retracing entered as separate dependent variables. In each model, age and gender were entered in Step 1, PPVT in Step 2, Raven in Step 3, forward digit span in Step 4, backward digit span in Step 5, and the two VSPT measures—agent-based VSPT and non-agent-based VSPT—in Step 6.

For directional approach, the full model accounted for 62.7% of the variance, *R*^2^ = 0.627, adjusted *R*^2^ = 0.567. Adding the two VSPT measures in the final step significantly improved model fit, Δ*R*^2^ = 0.051, Δ*F*(2, 50) = 3.43, *p* = 0.040. In the final model, agent-based VSPT significantly predicted directional approach, *β* = 0.339, *t* = 2.54, *p* = 0.014, whereas non-agent-based VSPT did not, *β* = −0.156, *t* = −1.15, *p* = 0.255. For route repetition, the full model accounted for 18.4% of the variance, *R*^2^ = 0.184, adjusted *R*^2^ = 0.053. Adding the two VSPT measures also significantly increased explained variance, Δ*R*^2^ = 0.114, Δ*F*(2, 50) = 3.49, *p* = 0.038. In the final model, non-agent-based VSPT significantly predicted route repetition, *β* = 0.529, *t* = 2.64, *p* = 0.011, whereas agent-based VSPT did not, *β* = −0.319, *t* = −1.62, *p* = 0.113. For route retracing, the full model accounted for 20.7% of the variance, *R*^2^ = 0.207, adjusted *R*^2^ = 0.077. Adding the two VSPT measures in the final step did not significantly improve model fit, Δ*R*^2^ = 0.002, Δ*F*(2, 49) = 0.08, *p* = 0.927. Neither agent-based VSPT, *β* = 0.065, *t* = 0.32, *p* = 0.754, nor non-agent-based VSPT, *β* = −0.069, *t* = −0.34, *p* = 0.738, significantly predicted route retracing (see Table 3, Figure 5).

**Table 3:** Standardized regression coefficients from the final hierarchical regression models predicting three components of wayfinding.

Predictor	Directional approach *β* [95% CI]	Route Repetition *β* [95% CI]	Route Retracing *β* [95% CI]
Age	0.020 [−0.165, 0.205]	0.011 [−0.265, 0.287]	−0.015 [−0.284, 0.254]
Gender	−0.132 [−0.324, 0.060]	0.044 [−0.240, 0.328]	−0.334^*^ [−0.615, −0.053]
PPVT	−0.221^†^ [−0.450, 0.008]	0.100 [−0.237, 0.437]	−0.330^†^ [−0.664, 0.004]
Raven	0.272^*^ [0.063, 0.481]	0.231 [−0.078, 0.540]	0.384^*^ [0.076, 0.692]
Forward digit span	−0.333^**^ [−0.578, −0.088]	−0.114 [−0.475, 0.247]	0.260 [−0.104, 0.624]
Backward digit span	0.360^**^ [0.140, 0.580]	−0.051 [−0.380, 0.278]	0.115 [−0.210, 0.440]
Agent-cued VSPT	0.339^*^ [0.070, 0.608]	−0.319 [−0.716, 0.078]	0.065 [−0.323, 0.453]
Non-agent VSPT	−0.156 [−0.428, 0.116]	0.529^*^ [0.126, 0.932]	−0.069 [−0.453, 0.315]
*R* ^2^	0.020 [−0.165, 0.205]	0.011 [−0.265, 0.287]	−0.015 [−0.284, 0.254]
Adjusted *R*^2^	−0.132 [−0.324, 0.060]	0.044 [−0.240, 0.328]	−0.334^*^ [−0.615, −0.053]

Values are standardized regression coefficients from the final step of each hierarchical regression model. Age and sex were entered as control variables in Step 1. PPVT was entered in Step 2, Raven in Step 3, forward digit span in Step 4, backward digit span in Step 5, and agent-cued and non-agent VSPT in Step 6. VSPT = visuospatial perspective taking; PPVT = Peabody Picture Vocabulary Test. All VIF values were below 3, indicating no serious multicollinearity. The hierarchical regression models included complete cases for all variables entered in each model, with valid Ns ranging from 52 to 59. ^†^*p* < 0.10. ^*^p < 0.05. ^**^p < 0.01.

**Figure 5:**
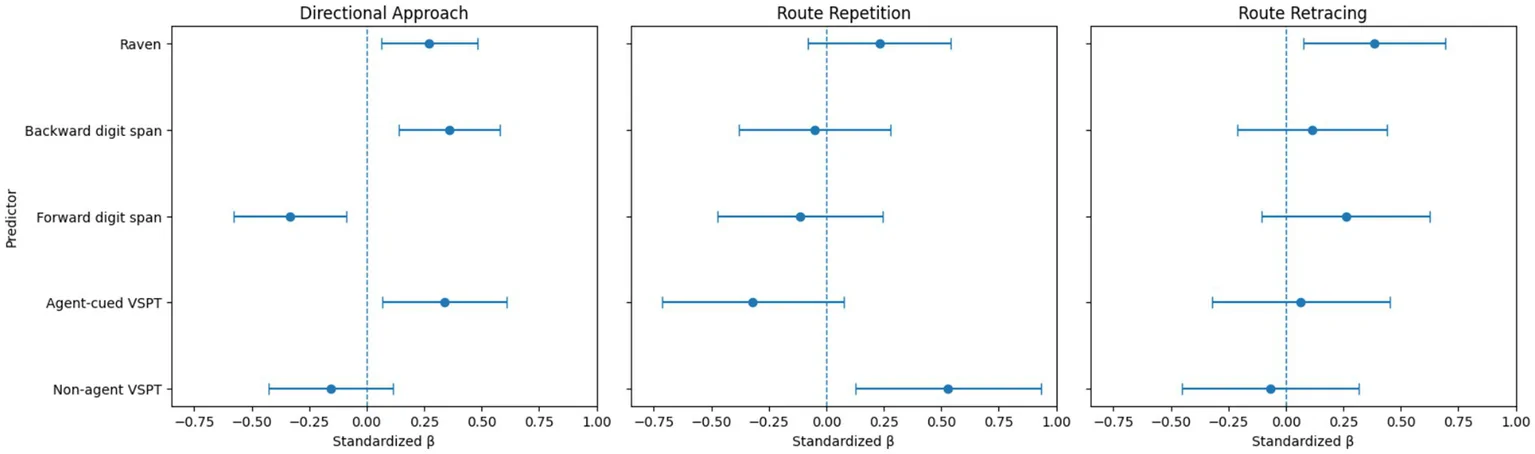
Key cognitive predictors across wayfinding tasks. Standardized regression coefficients for key cognitive predictors across the three wayfinding tasks. Points represent standardized *β* coefficients from the final hierarchical regression models; error bars represent 95% confidence intervals. Age, gender, and PPVT were included as covariates but are not displayed for clarity. VSPT, visuospatial perspective taking.

Overall, the final-step regression results indicated that the two VSPT measures contributed differently across wayfinding components: agent-based VSPT uniquely predicted directional approach, non-agent-based VSPT uniquely predicted route repetition, and neither VSPT measure predicted route retracing.

### Component-specific associations of working memory and nonverbal reasoning with wayfinding

To examine whether working memory and nonverbal reasoning showed component-specific associations with children’s wayfinding performance, separate hierarchical regression analyses were conducted for the three wayfinding tasks. In each model, predictors were entered sequentially: age and gender, PPVT, Raven’s Progressive Matrices, forward digit span, backward digit span, and agent-based and non-agent-based VSPT.

### Effects of nonverbal reasoning

The role of nonverbal reasoning, as measured by Raven’s Progressive Matrices, differed across the three tasks. For directional approach, adding Raven after controlling for age, gender, and PPVT significantly increased the explained variance, Δ*R*^2^ = 0.266, Δ*F*(1, 54) = 22.93, *p* < 0.001. In the final model, Raven remained a significant positive predictor of directional approach, *β* = 0.272, *t* = 2.62, *p* = 0.012.

For route repetition, adding Raven did not significantly improve the model, Δ*R*^2^ = 0.045, Δ*F*(1, 54) = 2.59, *p* = 0.113. Raven also did not significantly predict route repetition in the final model, *β* = 0.231, *t* = 1.50, *p* = 0.139.

For route retracing, the incremental effect of Raven was significant, Δ*R*^2^ = 0.091, Δ*F*(1, 53) = 5.72, *p* = 0.020. In the final model, Raven remained a significant positive predictor of route retracing, *β* = 0.384, *t* = 2.51, *p* = 0.016.

### Effects of working memory

The effects of forward and backward digit span were mainly observed for directional approach. After controlling for age, gender, PPVT, and Raven, forward digit span significantly increased the explained variance in directional approach, Δ*R*^2^ = 0.070, Δ*F*(1, 53) = 6.67, *p* = 0.013. Adding backward digit span further increased the explained variance, Δ*R*^2^ = 0.132, Δ*F*(1, 52) = 16.17, *p* < 0.001. In the final model, forward digit span significantly and negatively predicted directional approach, *β* = −0.333, *t* = −2.73, *p* = 0.009, whereas backward digit span significantly and positively predicted directional approach, *β* = 0.360, *t* = 3.28, *p* = 0.002.

For route repetition, neither forward nor backward digit span significantly increased the explained variance, Δ*R*^2^ = 0.003, *p* = 0.705, and Δ*R*^2^ = 0.011, *p* = 0.437, respectively. In the final model, neither forward digit span, *β* = −0.114, *p* = 0.528, nor backward digit span, *β* = −0.051, *p* = 0.757, was significant.

For route retracing, the incremental effects of forward and backward digit span were also nonsignificant, Δ*R*^2^ = 0.036, *p* = 0.131, and Δ*R*^2^ = 0.009, *p* = 0.463, respectively. In the final model, neither forward digit span, *β* = 0.260, *p* = 0.158, nor backward digit span, *β* = 0.115, *p* = 0.481, reached significance.

The incremental explained variance for each predictor block is presented in Table 4.

**Table 4:** Incremental variance explained by predictor blocks in hierarchical regression models predicting three components of wayfinding.

Outcome	PPVT Δ*R*^2^	Raven Δ*R*^2^	Forward digit span Δ*R*^2^	Backward digit span Δ*R*^2^	VSPT block Δ*R*^2^
Directional approach	0.096^*^	0.266^***^	0.070^*^	0.132^***^	0.051^*^
Route repetition	0.007	0.045	0.003	0.011	0.114^*^
Route retracing	0.026	0.091^*^	0.036	0.009	0.002

Values represent changes in *R*^2^ at each step of the hierarchical regression models. Age and sex were entered in Step 1, PPVT in Step 2, Raven in Step 3, forward digit span in Step 4, backward digit span in Step 5, and the VSPT block in Step 6. The VSPT block included both agent-cued VSPT and non-agent VSPT. PPVT, Peabody Picture Vocabulary Test; VSPT, visuospatial perspective taking. ^*^p < 0.05. ^***^p < 0.001.

### Overall summary

Taken together, the findings from RQ1–RQ3 indicate that wayfinding in 5- to 6-year-old children shows clear componential characteristics. First, the three wayfinding tasks differed significantly in difficulty, with the highest performance observed for route repetition, intermediate performance for directional approach, and the lowest performance for route retracing. Second, different forms of VSPT selectively predicted different navigation components: agent-based VSPT predicted directional approach, whereas non-agent-based VSPT predicted route repetition. Third, working memory and nonverbal reasoning also showed component-specific associations. Backward working memory was mainly associated with directional approach, whereas Raven’s Progressive Matrices primarily predicted directional approach and route retracing.

## Discussion

The present study examined performance differences and cognitive correlates across three wayfinding tasks in 5- to 6-year-old children. The results showed that route repetition, directional approach, and route retracing differed not only in mean performance, but also in their patterns of association with VSPT, working memory, and nonverbal reasoning. Overall, the findings are compatible with a componential view of children’s wayfinding, suggesting that route learning and use in early childhood may place partly different demands, ranging from reproducing a learned route to transforming directional relations and reconstructing a route from the reverse direction.

### Componential patterns in children’s wayfinding

First, The three wayfinding tasks showed a clear performance gradient: children performed best on route repetition, showed intermediate performance on directional approach, and performed lowest on route retracing. Although these tasks were adapted from Wiener et al.’s (2020) Navigation Test Suite and were therefore designed to differ in their spatial demands, the present findings indicate that these task differences were reflected, at least at the level of performance difficulty, in this child sample. Reproducing a learned route in the original direction appeared to be relatively accessible, whereas judging a previously encoded location from a new direction and retracing a route from the endpoint more challenging in terms of observed performance.

This performance pattern is broadly consistent with previous work showing that retracing or backtracking a route is more difficult than following a route in the original direction. Such difficulty is thought to arise because retracing requires individuals to go beyond simple landmark–action associations and use spatial relations more flexibly (Cornell et al., 1992). Adult studies have similarly suggested that route retracing involves more flexible use of spatial relations than route repetition, rather than simply requiring reuse of the originally learned route (Wiener et al., 2012, 2024). By applying this componential task framework to a child sample, the present study provides preliminary evidence that these tasks can be behaviorally differentiated in 5- to 6-year-old children.

Importantly, however, task difficulty differences alone do not demonstrate mechanism-level dissociation. A more compelling componential interpretation requires evidence that the tasks are associated with different cognitive predictors. The present results offer converging, although indirect, evidence for this interpretation: the three tasks differed in mean performance and showed different patterns of association with VSPT, working memory, and nonverbal reasoning. Thus, the performance gradient should be interpreted together with the regression findings, rather than as evidence of componential mechanisms on its own.

### Task-specific roles of VSPT

Second, agent-based VSPT primarily predicted directional approach, whereas non-agent-based VSPT primarily predicted route repetition. This pattern suggests that the relation between VSPT and wayfinding may not be global, but depends on the type of reference-frame transformation required by a given wayfinding task.

The association between agent-based VSPT and directional approach is theoretically meaningful. Directional approach requires children to judge a target location from a new approach direction, which resembles the need to reorganize spatial relations from an external position. The small figure used in the agent-based VSPT task may have provided children with a clear external bodily orientation, allowing them to translate the spatial judgment into the question of “where the target is from that position.” This interpretation is consistent with accounts of embodied perspective transformation and external viewpoint anchoring in VSPT research (Samuel et al., 2024).

This finding should not, however, be overinterpreted as evidence for a specifically social perspective-taking mechanism. Previous work suggests that the presence of a potential agent can alter the nature of spatial perspective taking by encouraging participants to adopt an embodied or externally anchored viewpoint (Clements-Stephens et al., 2013). At the same time, agent cues, directional cues, and interaction cues can all influence spatial perspective-taking performance. The effect of an avatar may therefore derive partly from the positional, orientational, and directional information it provides, rather than from social processing alone (Gunalp et al., 2019).

The predictive role of non-agent-based VSPT for route repetition is also informative. Although route repetition was the easiest of the three tasks, it is not simply a measure of sequential route memory. Even when reproducing a route in the original direction, children must continuously update the next action direction according to their current body orientation and environmental cues. Because non-agent-based VSPT does not provide an external bodily anchor, it may more closely capture children’s ability to perform spatial direction transformations and egocentric updating without explicit agent cues. Individual differences in route repetition may therefore be more closely associated with self-referenced directional updating than with transformation from an external agent’s perspective. This interpretation is broadly consistent with navigation research suggesting that some route-learning tasks can be supported by response-based or egocentric strategies that rely on sequential directional updating rather than flexible restructuring of spatial relations (Bullens et al., 2010).

### Componential roles of working memory and nonverbal reasoning

Third, working memory and nonverbal reasoning also showed task-specific associations. Backward digit span primarily predicted directional approach, but did not significantly predict route repetition or route retracing. This pattern suggests that directional approach may rely particularly on the manipulation and updating of information while spatial relations are being maintained. In this task, children must remember target–landmark relations and transform them into a new approach direction, making executive updating more relevant than in simple route reproduction.

Forward digit span negatively predicted directional approach. This result should be interpreted cautiously. Forward digit span primarily reflects verbal sequential maintenance, whereas backward digit span also involves information manipulation and updating (Baddeley, 2003). Forward digit span showed an unexpected negative association with directional approach. At present, the reason for this negative relation is unclear. It may reflect task-specific strategy differences, measurement instability, or sample-specific variation. Because the present study did not directly assess children’s strategy use, this finding should not be interpreted as evidence that a sequential memory strategy impairs wayfinding performance.

The findings for Raven’s Progressive Matrices suggest that nonverbal reasoning plays a more stable role in complex wayfinding components. Raven scores significantly predicted directional approach and route retracing, but did not independently predict route repetition. This pattern indicates that nonverbal reasoning is unlikely to be a general basis for all wayfinding tasks. Instead, it may be particularly relevant to tasks that require spatial-relational reorganization or transformation of route structure. Directional approach requires children to reorganize target–landmark relations from a new direction, whereas route retracing requires them to transform the original route into a return path. Both tasks go beyond simple forward route reproduction and may therefore place greater demands on relational reasoning and structural integration.

At the same time, Raven’s role should not be overinterpreted mechanistically. Raven’s Progressive Matrices provide a broad measure of nonverbal reasoning and may involve relational reasoning, rule detection, attentional control, and general fluid ability. Thus, the present findings indicate that the broader nonverbal reasoning ability indexed by Raven is reliably associated with more complex wayfinding components, but they do not show that Raven directly measures route reconstruction mechanisms. Future studies should include more specific tasks assessing spatial-relational reasoning, map-based reasoning, or route-structure reconstruction to test this interpretation more directly.

### The special status of route retracing

Route retracing deserves particular attention because it was the most difficult task but was not significantly predicted by either VSPT measure or by digit span. This pattern should not be taken to mean that retracing does not involve perspective transformation or working memory. Rather, it may indicate a mismatch between the cognitive demands of retracing and the measures used in the present study. The VSPT tasks assessed local, static perspective transformations, whereas route retracing required children to reorganize a learned route across multiple decision points while moving from the endpoint back to the start. Previous navigation research has similarly suggested that more flexible navigation tasks may depend less on simple egocentric response strategies and more on the ability to reorganize spatial relations across viewpoints and route segments (Broadbent et al., 2014). Similarly, digit span tasks primarily assess verbal sequence maintenance and general information manipulation, whereas retracing may require spatial sequence maintenance, path integration, or dynamic spatial updating. Future studies should include spatial span tasks, route-sequence memory tasks, and dynamic path-reversal tasks to examine these possibilities more directly.

### Theoretical contributions and limitations

The main contribution of this study is that it provides preliminary evidence for component-specific cognitive associations in children’s wayfinding. First, 5- to 6-year-old children showed different performance levels across route repetition, directional approach, and route retracing, suggesting that children’s wayfinding should not be represented by a single global score. Second, agent-based and non-agent-based VSPT were associated with different wayfinding components, suggesting that the relation between VSPT and navigation may depend on the type of viewpoint cue and reference-frame demand. Third, Raven’s Progressive Matrices and backward digit span were more strongly associated with the more complex wayfinding components, suggesting that flexible navigation may involve broader cognitive resources such as updating and relational reasoning. Together, these findings highlight the value of examining children’s wayfinding at the component level, while leaving open the causal and developmental mechanisms underlying the observed associations.

Several limitations should also be noted. First, the study used a cross-sectional correlational design, which does not allow causal conclusions about the effects of VSPT, working memory, or nonverbal reasoning on wayfinding. Second, the sample size was relatively limited, especially for hierarchical regression models that included multiple control variables; therefore, nonsignificant findings should be interpreted with caution. Third, the working memory measures were forward and backward digit span tasks, which do not fully capture spatial working memory. Fourth, the VSPT tasks used in this study mainly assessed local and static perspective transformations, whereas route retracing may involve route-level and dynamic direction updating. Finally, the study focused only on 5- to 6-year-old children. Future research should use longitudinal or cross-age designs to examine how these cognitive association patterns change with the development of spatial representations and executive functions. Although Little’s MCAR test did not indicate systematic missingness, the reduced sample size in the hierarchical regression models limited statistical power and may have increased the uncertainty of some estimates. Accordingly, nonsignificant regression findings should be interpreted cautiously and require replication in larger samples with more complete data.

## Conclusion

The present study provides evidence that wayfinding in 5- to 6-year-old children may show early signs of componential organization. Route repetition, directional approach, and route retracing differed not only in task difficulty but also in their cognitive correlates. Agent-based was most clearly associated with directional approach, whereas non-agent-based VSPT was most clearly associated with route repetition. Backward digit span was associated primarily with directional approach, whereas Raven’s Progressive Matrices predicted both directional approach and route retracing. Overall, these findings suggest that early wayfinding development is not simply a matter of improved route memory; instead, different wayfinding components may place partly different demands on cognitive resources, including perspective transformation, working-memory updating, and relational reasoning.

## Data Availability

The raw data supporting the conclusions of this article will be made available by the authors, without undue reservation.
